# PINK1/Parkin‐mediated mitophagy enhances the survival of *Staphylococcus aureus* in bovine macrophages

**DOI:** 10.1111/jcmm.17664

**Published:** 2023-01-09

**Authors:** Xi Zhou, Kangjun Liu, Jianji Li, Luying Cui, Junsheng Dong, Jun Li, Xia Meng, Guoqiang Zhu, Heng Wang

**Affiliations:** ^1^ Jiangsu Co‐innovation Center for Prevention and Control of Important Animal Infectious Diseases and Zoonoses, College of Veterinary Medicine Yangzhou University Yangzhou China; ^2^ Joint International Research Laboratory of Agriculture and Agri‐product Safety of the Ministry of Education Yangzhou China

**Keywords:** bovine macrophages, mitophagy, PINK1, *Staphylococcus aureus*

## Abstract

Mitochondria are cellular organelles that are involved in various metabolic processes, and damage to mitochondria can affect cell health and even lead to disease. Mitophagy is a mechanism by which cells selectively wrap and degrade damaged mitochondria to maintain cell homeostasis. However, studies have not focused on whether mitophagy is involved in the occurrence of *Staphylococcus aureus* (*S. aureus*)‐induced mastitis in dairy cows. Here, we found that *S. aureus* infection of bovine macrophages leads to oxidative damage and mitochondria damage. The expression of LC3, PINK1 and Parkin was significantly increased after intracellular infection. We observed changes in the morphology of mitochondria and the emergence of mitochondrial autolysosomes in bovine macrophages by transmission electron microscopy and found that enhanced mitophagy promoted bacterial proliferation in the cell. In conclusion, this study demonstrates that *S. aureus* infection of bovine macrophages induces mitophagy through the PINK1/Parkin pathway, and this mechanism is used by the bacteria to avoid macrophage‐induced death. These findings provide new ideas and references for the prevention and treatment of *S. aureus* infection.

## 1 INTRODUCTION

Mastitis is one of the great challenges facing farms today because it leads to harm and losses to livestock farms in many aspects, such as decreased milk yield and milk quality, increased treatment cost and accelerated elimination rates. *Staphylococcus aureus* (*S. aureus*) is a widely studied gram‐positive bacterium that causes mastitis in animals, and it is among the most common pathogens underlying mastitis.^1^, ^2^ *S. aureus* is an intracellular bacteria of a variety of cells, including epithelial cells, neutrophils and macrophages.^3^, ^4^, ^5^ Because of its ability to colonize cells, the bacterium persists within herds and causes infection.^6^ Antibiotics are still widely used for mastitis treatment. However, the abuse of antibiotics will lead to the emergence of drug‐resistant strains of *S. aureus*.^7^, ^8^ When the antibiotic pressure is removed, *S. aureus* escapes from the cell and replicates, thus infecting neighbouring cells. Therefore, it is necessary to find new therapeutic strategies to overcome *S. aureus* infection.

Effective microbial clearance by macrophages depends on phagocytosis and phagolysosomal killing mediated by oxidative bursts, acidification, and degradative enzymes.^9^ Macrophages can directly destroy most invading bacteria through the phagolysosomal pathway.^10^ However, some pathogenic microorganisms, including some drug‐resistant microorganisms, have evolved sophisticated mechanisms to prevent phagocytosis or escape intracellular degradation. The survival strategy of the pathogen varies based on the strain and host cell type. *S. aureus* can escape the phagosomes of professional and nonprofessional phagocytes and disrupt autophagy, thereby inducing apoptosis and even antiapoptotic programmes.^11^ Studies have shown that *S. aureus* can evade immune clearance by adhering to and infiltrating mammary epithelial cells.^12^, ^13^ After entering cells, *S. aureus* can inhibit lysosome function to avoid autophagy clearance.^3^ However, *S. aureus* can promote its intracellular survival by blocking autophagic flux in dairy cow macrophages.^14^

Fission‐fusion imbalance and mitochondrial dysfunction have been observed in many pathological processes, and the interaction between mitochondria and pathogenic bacteria has been widely reported.^15^, ^16^, ^17^ Mitophagy is a selective autophagy that can degrade damaged or excessive mitochondria in cells and regulate cell homeostasis. The PINK1/Parkin axis is a major pathway that mediates mitophagy and has been widely studied.^18^, ^19^, ^20^ Notably, both gram‐negative bacteria, such as *Escherichia coli* toxin LPS, and gram‐positive bacteria, such as *Staphylococcus epidermidis*, can induce PINK1‐mediated mitophagy in cells.^21^, ^22^ Here, we infected bovine macrophages with *S. aureus* and found that the PINK1/Parkin‐mediated mitophagy pathway was activated. Thus, mitophagy interventions could affect the survival of intracellular bacteria.

## 2 MATERIALS AND METHODS

### 2.1 Reagents

Fetal bovine serum (FBS) and other tissue culture reagents were purchased from Gibco BRL Co. Dulbecco's modified Eagle's medium/F12 (DMEM/F12) and carbonyl cyanide m‐chlorophenylhydrazone (CCCP) were purchased from Sigma Aldrich. MitoSOX Red, LysoTracker Green, MitoTracker Red and Lipofectamine 3000 were obtained from Invitrogen Life Technologies. Anti‐GAPDH, anti‐TOM20 and horseradish peroxidase (HRP)‐conjugated goat anti‐rabbit IgG were supplied by Cell Signalling Technology. Anti‐TIM23, anti‐Drp1 and anti‐Mfn1 were from Santa Cruz Biotechnology, Inc. Anti‐LC3 and HRP‐conjugated goat anti‐mouse IgG were acquired from Medical & Biological Laboratories Co. Ltd. Anti‐HSP60 and anti‐PINK1 were from Abcam. Anti‐COX IV was purchased from Novus Biologicals. Mitochondrial division inhibitor 1 (Mdivi‐1) was supplied by MedChem Express.

### 2.2 Preparation of *S. aureus*

*Staphylococcus aureus* (ATCC 29213) was selected and cultured overnight at 37°C in 20 ml of liquid Luria‐Bertani (LB). When *S. aureus* reached the logarithmic growth phase, it was washed three times with phosphate‐buffered saline (PBS) and then diluted to a certain bacterial concentration (multiplicity of infection = 1:1) with DMEM‐F12 medium.

### 2.3 Culture and treatment of bovine macrophages

**FIGURE 1:**
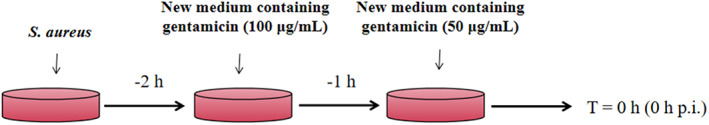
The intracellular infection model of *S. aureus*. Bovine macrophages were cocultured with *S. aureus* for 2 h and then cultured in DMEM‐F12 medium supplemented with 100 μg/ml gentamicin sulfate for 1 h to kill extracellular bacteria. This medium was subsequently replaced with fresh medium containing 50 μg/ml gentamicin sulfate, and this event was considered time zero (T = 0 h p.i.)

The bovine macrophage cell line was generously provided by Professor Aizhen Guo from the Huazhong Agricultural University of Wuhan, China. Bovine macrophage cells were cultured in DMEM‐F12 supplemented with 10% FBS at 37°C with 5% CO_2_. The intracellular infection model was treated as follows based on previous studies^14^, ^23^ (Figure 1): bovine macrophages were cocultured with *S. aureus* for 2 h and then cultured in DMEM‐F12 medium supplemented with 100 μg/ml gentamicin sulfate for 1 h to kill extracellular bacteria. This medium was subsequently replaced with fresh medium containing 50 μg/ml gentamicin sulfate, and this event was considered time zero (T = 0 h p.i.).

### 2.4 Total ROS detection

2′, 7′‐Dichlorofluorescin diacetate (DCFH‐DA) was used to detect ROS generation in this study. In short, bovine macrophages were intracellularly infected with *S. aureus* for 0, 2 and 4 h. Subsequently, the cells were collected and suspended in 10 μM DCFH‐DA and incubated in an incubator at 37°C for 20 min. The green fluorescence intensity at 500/525 nm was detected by flow cytometry.

### 2.5 Mitochondrial ROS assay

Methodology based on Kaufmann et al.^24^ The infected macrophages were digested with trypsin and collected. The cells were suspended in 1 μM MitoSOX and incubated in a 37°C water bath for 20–40 min. Warm PBS was used to wash the cells twice, and then the cells were suspended. Flow cytometry was used to measure the fluorescence intensity at 510/580 nm.

### 2.6 Mitochondrial membrane potential assays

Tetraethylbenzimidazolylcarbocyanine iodide (JC‐1) was used to measure the mitochondrial membrane potential (MMP) in this study. Briefly, bovine macrophages were harvested at T = 0, 2, and 4 h p.i. The cells were stained with JC‐1 staining solution, fully mixed and incubated in an incubator at 37°C for 20 min. Afterward, the cells were washed twice with JC‐1 staining buffer and resuspended in 1 ml JC‐1 staining buffer. Both the red (514/529 nm) and green (585/590 nm) fluorescence intensities were detected by flow cytometry.

### 2.7 Transmission electron microscopy

Infected bovine macrophages were collected and fixed with 2.5% glutaraldehyde at 4°C overnight. Subsequently, the cell masses were fixed with 1% citric acid at 4°C for 2 h, dehydrated in gradient ethanol and 100% acetone, and then polymerized after embedding in resin. Ultrathin sections (70 nm) were prepared and stained. Finally, the sections were observed by transmission electron microscopy (HITACHI 7800, Japan).

### 2.8 Western blot analysis

The cells were harvested and lysed with radioimmunoprecipitation assay buffer containing protease inhibitor and protein phosphatase inhibitor, and total protein was obtained from the supernatant. The protein concentration was determined by a bicinchoninic acid protein assay kit (Beyotime). The proteins were isolated by SDS–PAGE and transferred to polyvinylidene fluoride (PVDF) membranes (Millipore). The LC3 membrane was blocked overnight with Tris‐buffered saline and Tween‐20 (TBST) containing 10% skim milk at 4°C. Other membranes were blocked with TBST containing 5% skim milk at room temperature for 1 h. Then, they were incubated with a specific primary antibody at 4°C overnight. All membranes were fully washed with TBST and incubated with secondary antibody at room temperature for 1.5 h. All bands were detected by enhanced chemiluminescence (ECL) and analysed by ImageJ (NIN).

### 2.9 Immunofluorescence

Cells were seeded on glass coverslips. The cells were infected with *S. aureus*, fixed with 4% paraformaldehyde at T = 2 h p.i., and then permeabilized with Triton X‐100. Blocking was performed in 5% BSA in PBS for 1 h at room temperature. Coverslips were incubated with anti‐LC3 and anti‐HSP60 antibodies for 1 h at room temperature and then washed three times with PBS. Fluorescent secondary antibodies were used to incubate the coverslips at room temperature for 1 h. Finally, the cells were stained with DAPI. After washing three times with PBS, all samples were visualized with a confocal fluorescence microscope (Leica TCS SP8 STED, Leica Corp).

### 2.10 Fluorescence colocalization

The cells were inoculated in a confocal laser dish. When the cells reached confluency, 200 nM MitoTracker was incubated with the macrophages for 30 min and then the appropriate treatment was administered. The cells were then incubated with 200 nM LysoTracker for 30 min. Finally, confocal fluorescence microscopy was used to observe colocalization.

### 2.11 RNA interference of PINK1

According to the manufacturer's instructions, 50 nM PINK1‐targeting small interfering RNA (siRNA‐PINK1) or nonspecific control siRNA (NC‐siRNA) was used to transfect the cells using Lipofectamine 3000. At 48 h following transfection, the cells were infected with *S. aureus*.

### 2.12 Bacterial intracellular survival

Bovine macrophages were pretreated with 10 μM mitophagy inhibitor Mdivi‐1 and 20 μM mitophagy inducer CCCP for 2 h and then infected with *S. aureus*. At T = 2 h p.i. The cells were lysed with 0.3% Triton X‐100 (in PBS) for 10 ~ 15 min. The cell lysates were diluted at appropriate multiples and inoculated on LB agar plates. After 10 h, the colonies were counted to evaluate the colony forming units (CFUs) of the intracellular bacteria.

### 2.13 Statistical analysis

All data represent the mean ± SEM from three independent repeat experiments. Statistical analyses were performed using the IBM SSPS 17.0 package (SPSS Inc./IBM Corp.). Significance was identified by independent‐samples *t* test or one‐way anova and least significant difference test, and *p* < 0.05 was accepted as significant.

## 3 RESULT

### 3.1 Intracellular *S. aureus* alters the mitochondrial morphology and function of bovine macrophages

**FIGURE 2:**
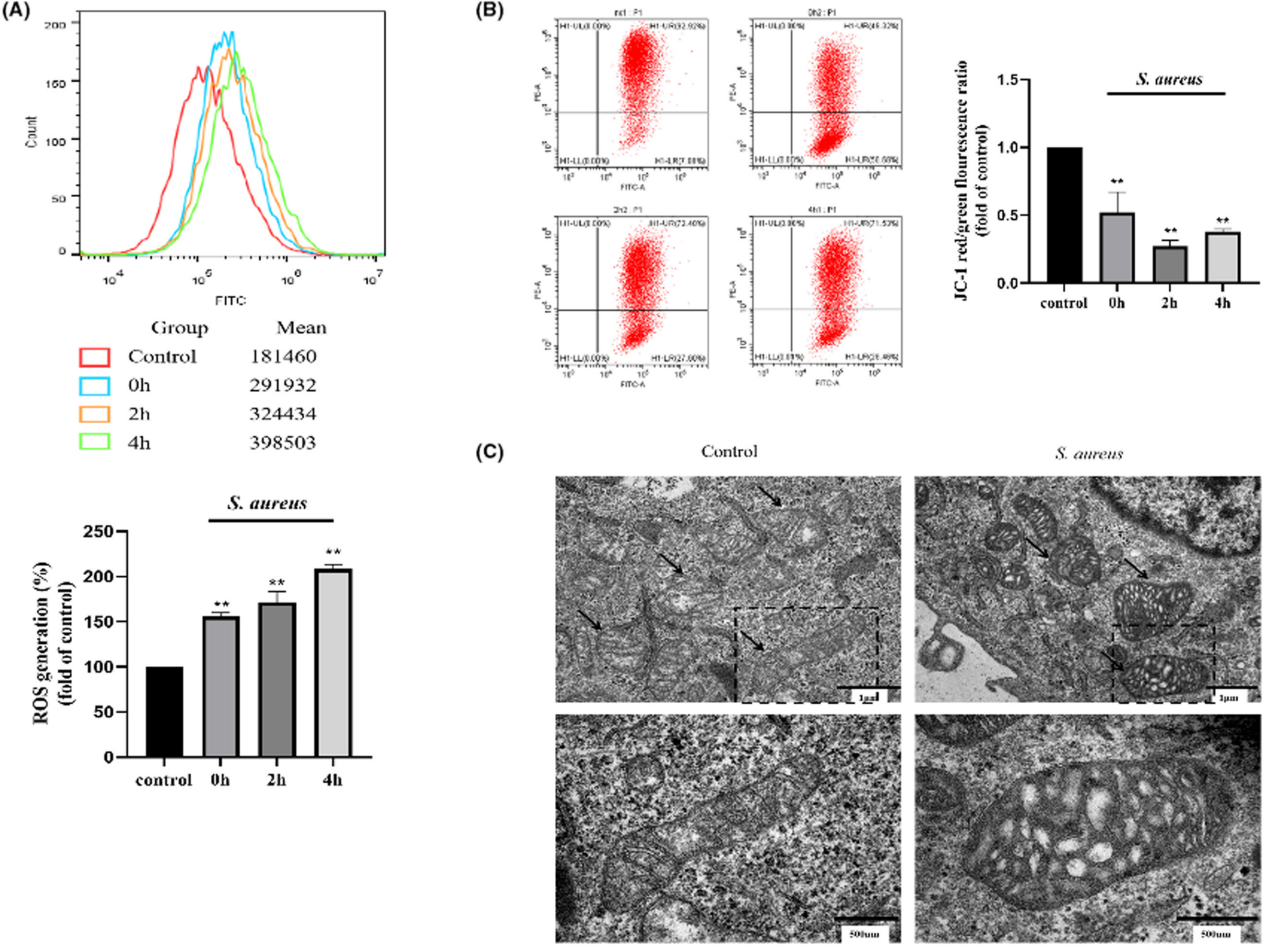
*S. aureus* affects bovine macrophage mitochondria. (A) Changes in total ROS in cells at 0 h, 2 h and 4 h. (B) Changes in mitochondrial membrane potential in cells at 0 h, 2 h and 4 h. (C) Observation of mitochondria under transmission electron microscopy at T = 2 h p.i. The data are presented as the means ± SEM. Each experiment was repeated three times. Significance was identified by independent‐samples *t* test or one‐way anova and least significant difference test, and *p* < 0.05 was accepted as significant. **p < 0.01 vs. untreated control group

To explore whether *S. aureus* infection can cause mitochondrial damage, we first detected changes in ROS production in macrophages and found that the production of total ROS in cells increased with prolonged infection time (*p* < 0.01) (Figure 2A). At the same time, a significant decrease in MMP was detected (*p* < 0.01) (Figure 2B). In addition, morphological changes in mitochondria were observed by transmission electron microscopy (Figure 2C). In normal cells, the morphology of mitochondria was intact and the structure of the bilayer membrane and cristae was clearly visible. However, in infected cells, the mitochondria were swollen and vacuolated, the membrane structure was indistinct, cristae cannot be seen, and fragments of broken mitochondria are occasionally visible. Overall, we confirmed that intracellular infection with *S. aureus* resulted in mitochondrial damage to bovine macrophages.

### 3.2 Intracellular *S. aureus* induced mitophagy in bovine macrophages

**FIGURE 3:**
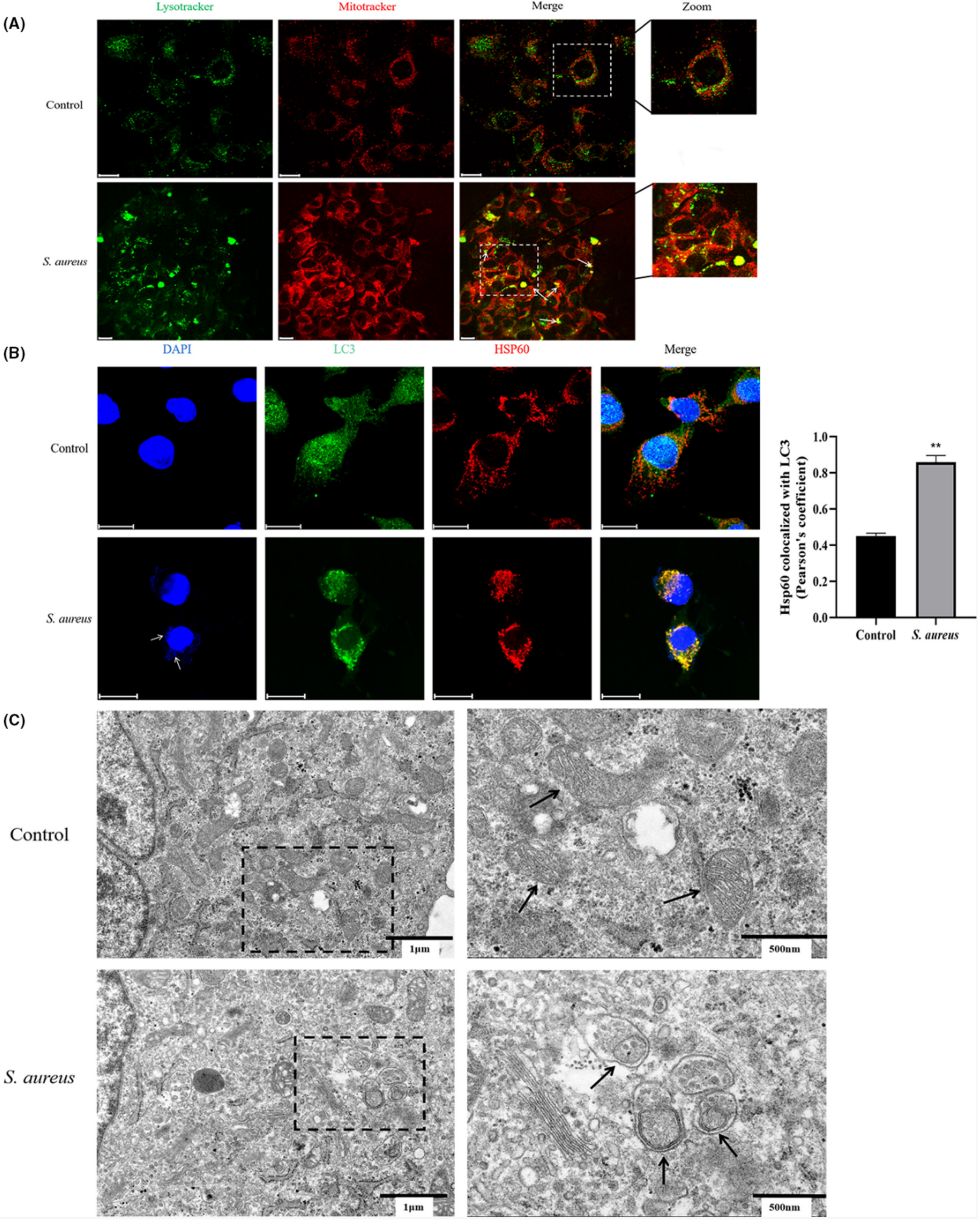
Mitophagy developing in *S. aureus* infected bovine macrophages. (A) Observation of colocalization of mitochondria (red) and lysosomes (green) under confocal microscopy. (B) Observation of colocalization of LC3 (green) and HSP60 (red) under confocal microscopy and quantitative statistics by Pearson's coefficient. White arrows point to *S. aureus*. (C) Observation of mitochondria engulfed by lysosomes under transmission electron microscopy. Black arrows point to mitochondria. Scale bar = 20 μm for A and B. The data are presented as the means ± SEM. Each experiment was repeated three times. Significance was identified by independent‐samples *t* test or one‐way anova and least significant difference test, and *p* < 0.05 was accepted as significant. **p < 0.01 vs. untreated control group

After *S. aureus* infection at T = 2 h p.i., the colocalization of mitochondria and lysosomes was observed by laser confocal microscopy (Figure 3A) and the colocalization of LC3 and HSP60 was also enhanced (*p* < 0.01) (Figure 3B). This result suggested that both the early and late stages of mitophagy in bovine macrophages were induced by *S. aureus* and the level of mitophagy in cells increased. Finally, we observed autolysosomes by transmission electron microscopy (Figure 3C). In this process, mitochondria are engulfed by lysosomes, which are wrapped by a monolayer membrane, and the bilayer structure of mitochondria can be seen inside.

### 3.3 Intracellular *S. aureus* activates PINK1/Parkin pathway mitophagy

**FIGURE 4:**
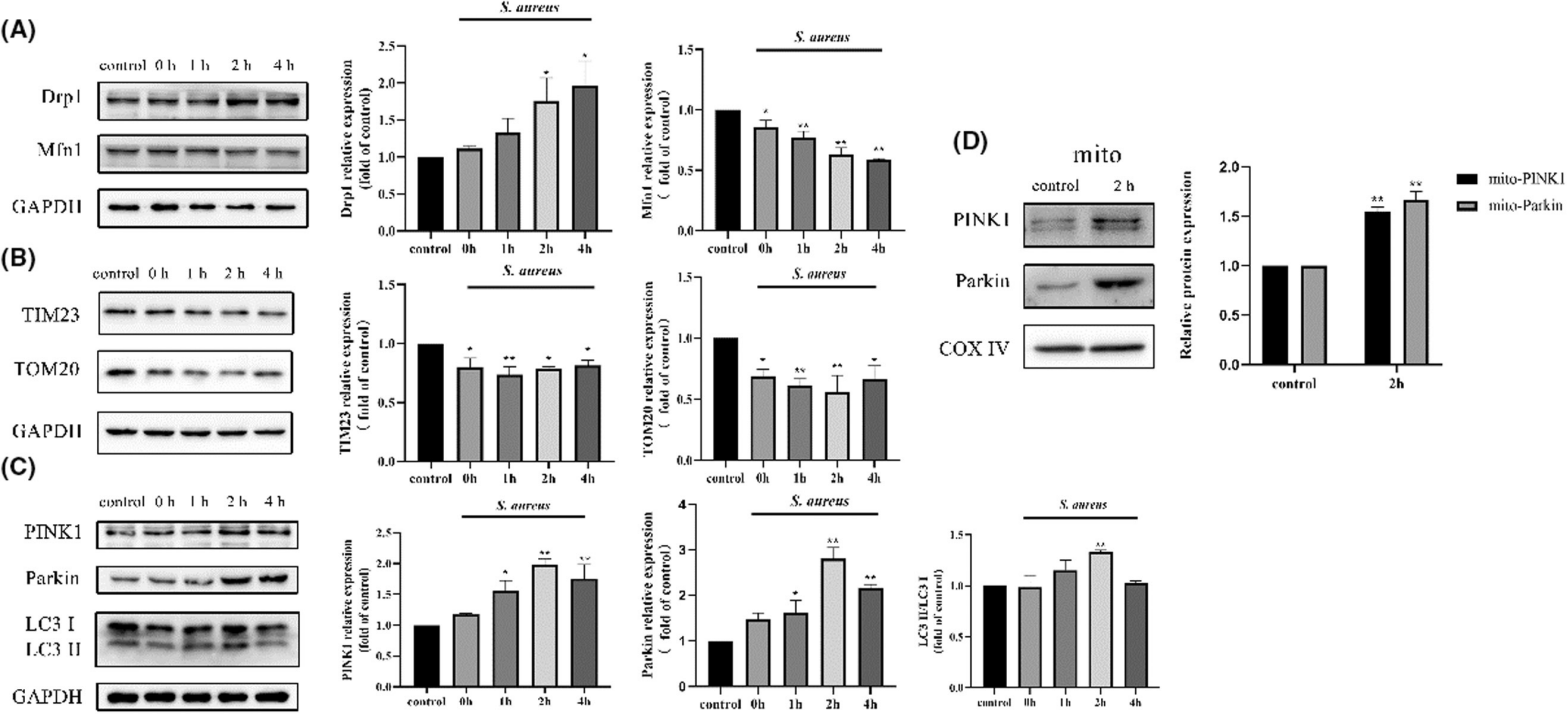
PINK1‐Parkin mediated mitophagy was activated by *S. aureus* in bovine macrophages. (A) The expression of mitochondrial dynamics related proteins Drp1 and Mfn1 at time point 0 h, 1 h, 2 h and 4 h. (B) The expression of mitochondrial membrane related proteins TIM23 and TOM20 at time point 0 h, 1 h, 2 h and 4 h. (C) The expression of mitophagy related proteins PINK1, Parkin and LC3 at time point 0 h, 1 h, 2 h and 4 h. (D) The expression of PINK1 and Parkin in mitochondria proteins at time point 2 h. The data are presented as the means ± SEM. Each experiment was repeated 3 times. Significance was identified by independent‐samples *t* test or one‐way anova and least significant difference test, and *p* < 0.05 was accepted as significant. *p < 0.05 vs. untreated control group, **p < 0.01 vs. untreated control group

Furthermore, we studied the influence of *S. aureus* intracellular infection on PINK1/Parkin. By Western blotting, we found that the expression of the mitochondrial fission‐related protein Drp1 was significantly increased at different time points (*p* < 0.05) (Figure 4A) while the expression of the mitochondrial fusion protein Mfn1 was decreased (*p* < 0.05 or 0.01) (Figure 4A). *S. aureus* also caused a decrease in the expression of the mitochondrial membrane proteins TOM20 and TIM23 in bovine macrophages at different time points (*p* < 0.05 or 0.01) (Figure 4B). The relative expression of the autophagy protein LC3 increased at T = 2 h p.i. (*p* < 0.05) (Figure 4B). PINK1 and Parkin expression among the total protein content was upregulated by *S. aureus* (*p* < 0.05 or 0.01) (Figure 4C), and these changes were more obvious for mitochondrial proteins (*p* < 0.01) (Figure 4D). Collectively, the changes in these proteins suggest that *S. aureus* infection altered the mitochondrial dynamics in bovine macrophages, which resulted in mass loss, and activated the PINK1/Parkin pathway.

### 3.4 Intracellular *S. aureus*‐induced mitophagy is PINK1‐dependent in bovine macrophages

**FIGURE 5:**
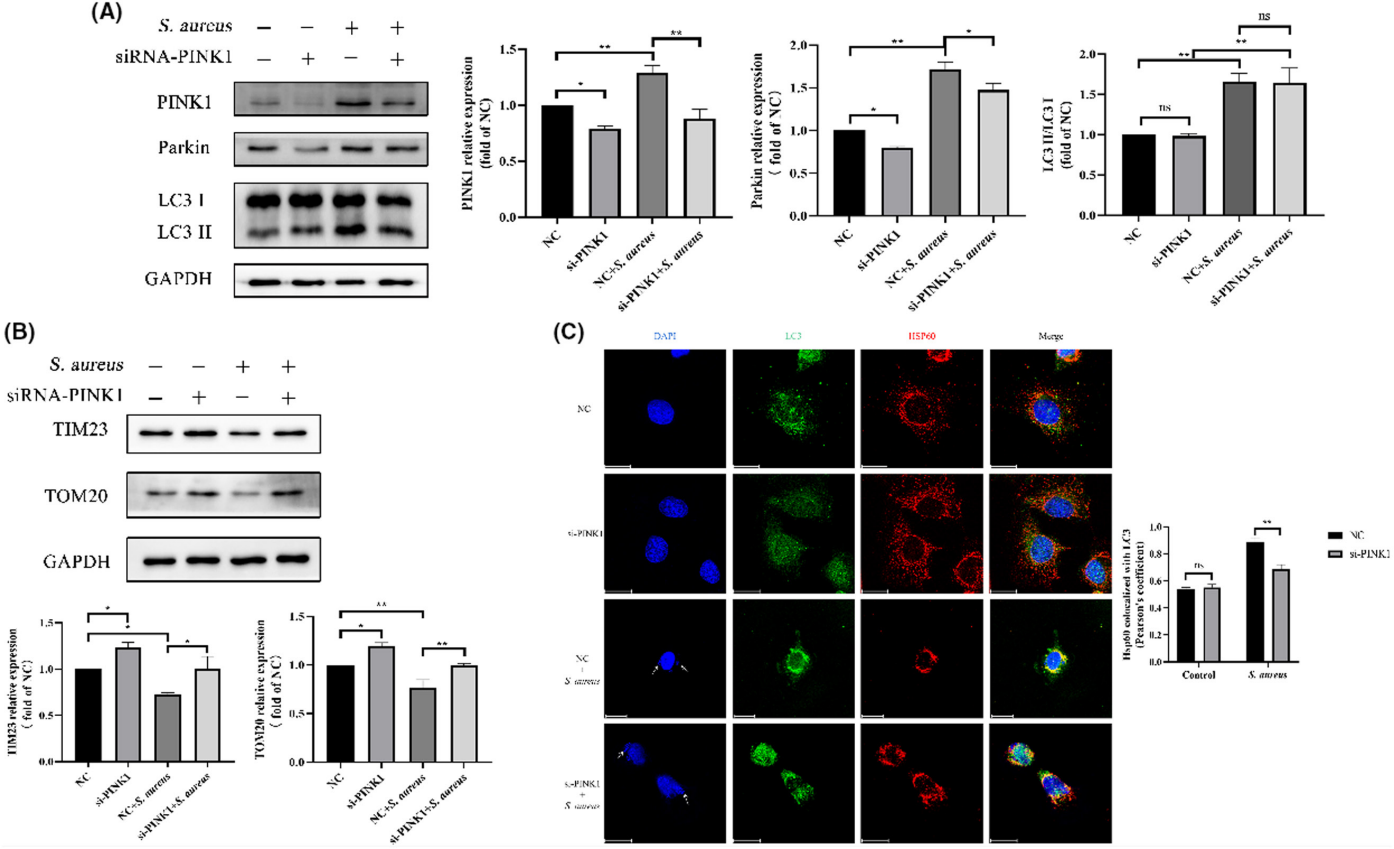
Mitophagy is PINK1‐Parkin dependent in bovine macrophages induced by intracellular *S. aureus*. 50 nM siRNA‐PINK1 or NC‐siRNA was used. (A) PINK1, Parkin and LC3 levels in cells infected with *S. aureus* in the presence or absence of siRNA‐PINK1. (B) TIM23 and TOM20 levels in cells infected with *S. aureus* in the presence or absence of siRNA‐PINK1. (C) Colocalization of LC3 (green) and HSP60 (red) in cells infected with *S. aureus* in the presence or absence of siRNA‐PINK1 and quantitative statistics by Pearson's coefficient. White arrows point to *S. aureus*. Scale bar = 20 μm. The data are presented as the means ± SEM. Each experiment was repeated three times. Significance was identified by independent‐samples *t* test or one‐way anova and least significant difference test, and *p* < 0.05 was accepted as significant. *p < 0.05, **p < 0.01

To further investigate the role of the PINK1‐Parkin pathway in *S. aureus* intracellular infection, siRNA‐PINK1 was used to interfere with the expression of PINK1 in bovine macrophages. As expected, the PINK1 protein elevation induced by *S. aureus* was reversed by siRNA‐PINK1 at T = 2 h p.i. (*p* < 0.01) (Figure 5A). The change in Parkin was consistent with this finding (*p* < 0.05) (Figure 5A). Moreover, the decline in TOM20 and TIM23 was also reversed by siRNA‐PINK1 (*p* < 0.05 or 0.01) (Figure 5B), which also blocked the strong colocalization of LC3 and HSP60 (*p* < 0.01) (Figure 5C). However, the relative expression level of LC3 did not change significantly (*p >* 0.05) (Figure 5A). In general, inhibition of PINK1 expression can reverse the mitophagy induced by intracellular *S. aureus*.

### 3.5 Regulation of mitophagy affects bacterial load

**FIGURE 6:**
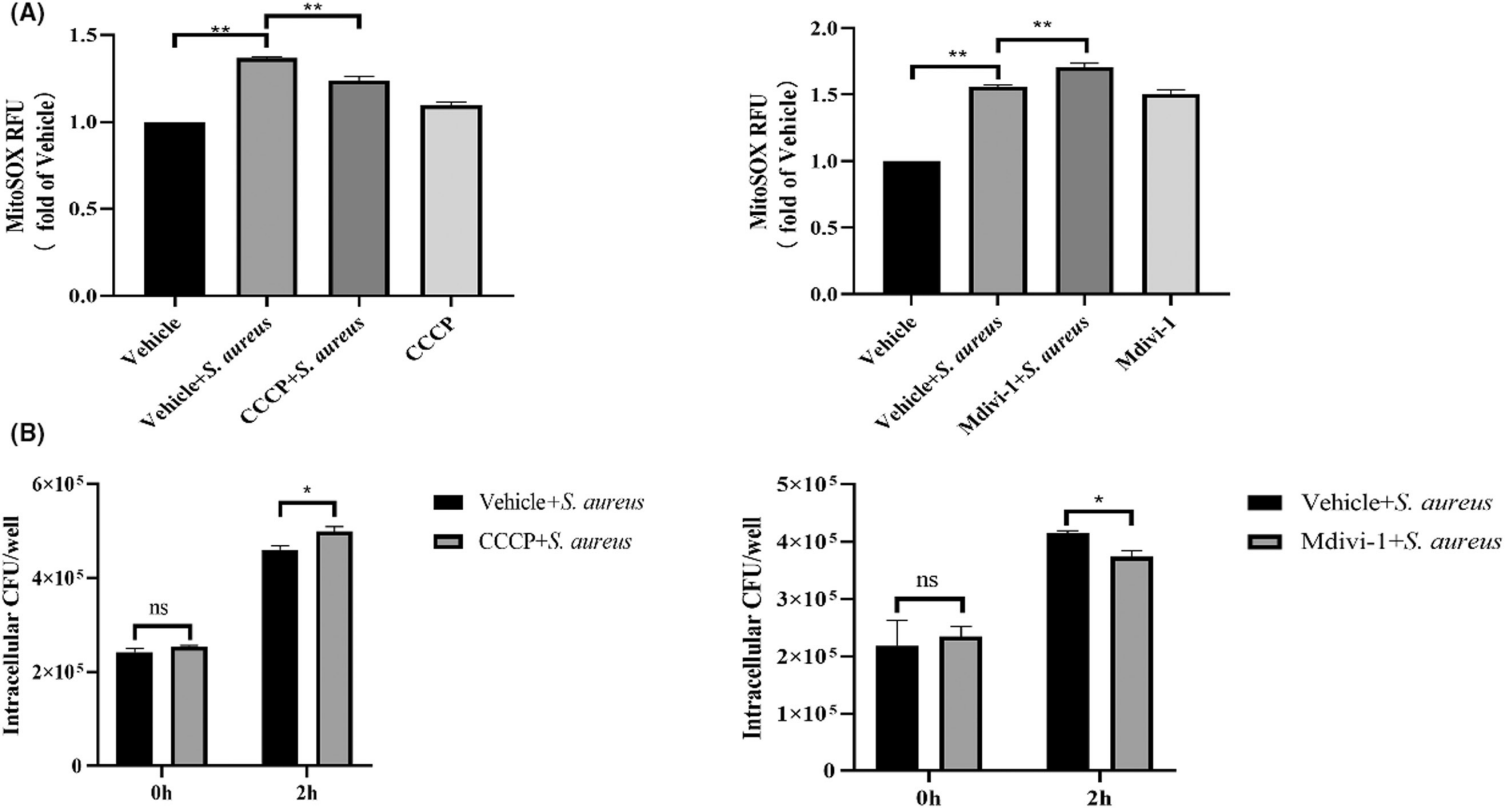
MtROS and *S. aureus* load of infected bovine macrophages were affected by mitophagy regulator. 10 μM Mdivi‐1 and 20 μM CCCP were used. (A) Effects of mitophagy regulators on mtROS. (B) Effects of mitophagy regulators on *S. aureus* load. The data are presented as the means ± SEM. Each experiment was repeated three times. Significance was identified by independent‐samples *t* test or one‐way anova and least significant difference test, and *p* < 0.05 was accepted as significant. *p < 0.05, **p < 0.01

Mitochondrial depolarization due to environmental stress such as CCCP, a mitochondrial oxidative phosphorylation uncoupler, induces mitophagy‐mediated clearance of damaged mitochondria or activates apoptosis. Drp1 is involved in the mitochondrial fission mechanism, and activated Drp1 can stimulate the fission process in mitophagy. Mdivi‐1, an inhibitor of Drp1, was reported in studies to block mitochondrial fission. CCCP and Mdivi‐1 have been reported in several studies as regulators of mitophagy.^25^, ^26^, ^28^ Next, we focused on the relationship between mitochondria and intracellular parasitism of *S. aureus*. CCCP and Mdivi‐1 were used to regulate mitochondrial autophagy. As observed, the CCCP treatment partially reversed the increase in mitochondrial ROS (mtROS) induced by *S. aureus* (*p* < 0.01) (Figure 6A), and an increase in intracellular bacterial load was detected (*p* < 0.05) (Figure 6B). In contrast, Mdivi‐1, an inhibitor of mitophagy, increased the mtROS content (*p* < 0.01) (Figure 6A) and reduced (*p* < 0.01) the intracellular *S. aureus* levels (Figure 6B). Notably, the bidirectional regulation of mitophagy produced completely different results, suggesting the importance of mitophagy in the intracellular infection of bacteria.

### 3.6 Inhibition of the PINK1/Parkin pathway affects *S. aureus* load

**FIGURE 7:**
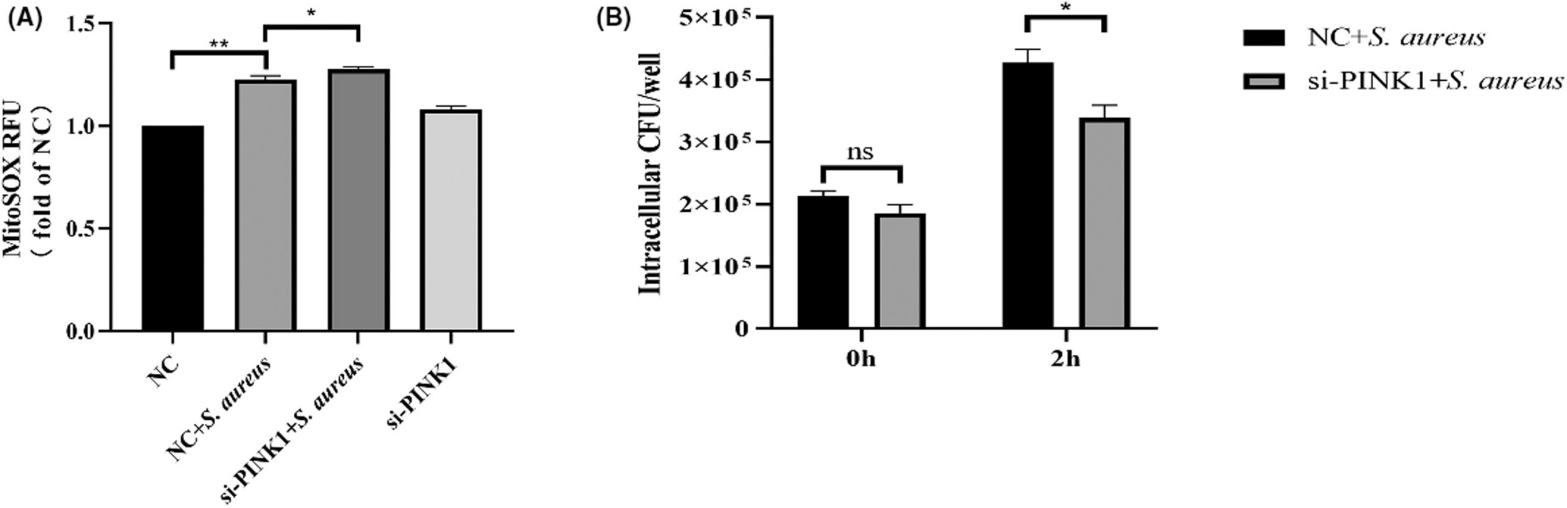
MtROS and *S. aureus* load of infected bovine macrophages were affected by siRNA‐PINK1. (A) Effects of mitophagy regulators on mtROS. (B) Effects of mitophagy regulators on *S. aureus* load. The data are presented as the means ± SEM. Each experiment was repeated three times. Significance was identified by independent‐samples *t* test or one‐way anova and least significant difference test, and *p* < 0.05 was accepted as significant. *p < 0.05, **p < 0.01

As previously mentioned, the mtROS level and bacterial load in bovine macrophages are affected by the regulation of mitochondrial autophagy. Therefore, we hypothesized that accurate regulation of the PINK1/Parkin pathway would also alter these indicators. The results were similar to that for Mdivi‐1‐treated cells and showed that after interfering with PINK1 expression, *S. aureus* induced greater mtROS production (*p* < 0.05) (Figure 7A) and less intracellular CFUs (*p* < 0.05) (Figure 7B) compared with the NC‐siRNA group. Taken together, we found that inhibiting the activation of the PINK1/Parkin pathway can reduce the *S. aureus* load.

## 4 DISCUSSION

*Staphylococcus aureus* has attracted increasing attention because of its pathogenicity, drug resistance and immune evasion mechanism. In this study, we found that *S. aureus* can utilize mitophagy to proliferate in cells.

*Staphylococcus aureus* is a facultative intracellular parasitic bacterium, and *S. aureus*‐induced dairy mastitis is difficult to prevent and treat in production due to the infectivity of *S. aureus* and its ability to hide in cells; thus, this illness causes inestimable economic losses to the global dairy industry. *S. aureus* infection of bovine mammary glands usually occurs in three stages: (1) adhesion to the nipple skin and subcutaneous matrix; (2) invasion and physical destruction of breast tissue and cells; and (3) evasion of the host immune system and proliferation in cells and tissues. According to research, more than 20 molecules are associated with *S. aureus* immune evasion.^27^ Consequently, exploring the complex immune escape mechanism of *S. aureus* is an important direction for the effective prevention and treatment of *S. aureus* mastitis.

Mitochondria are important organelles for oxidation and ATP production in cells. The intricate pathogenicity of *S. aureus* leads to strategies that target host cell mitochondria to dismantle and disrupt the immune response, which ultimately allow the bacteria to spread.^16^ Previous studies have partially revealed the relationship between mitochondria, ROS and intracellular bacteria.^15^, ^17^, ^28^ In this experiment, the total ROS level increased significantly after *S. aureus* intracellular infection of bovine macrophages. At the same time, MMP decreased significantly. These results indicate that the physiological processes of mitochondria have been disrupted. Elevated ROS levels can cause the opening of mitochondrial permeability transition pores (mPTP), after which mitochondria depolarize and MMP decreases.^29^ Damaged mitochondrial present oedema and may even collapse,^30^ resulting in mitochondrial swelling and structural blurriness as well as a reduction in TIM23 and TOM20,^20^, ^28^ as we observed. In turn, the damaged structure and function of mitochondria further aggravated the increase in ROS production, thus forming a vicious cycle and finally leading to cell death.^31^

We have shown that mitochondria are damaged during infection, and we sought to determine whether mitophagy occurs in cells. Our results confirmed that *S. aureus* induced mitophagy by detecting the colocalization of LC3 with HSP60 and mitochondria with lysosomes. More importantly, we found that mitochondria were engulfed by lysosomes.

PINK1/Parkin is a classic mitophagy pathway that was first found to be closely associated with Parkinson's disease in humans; however, growing evidence indicates that this pathway plays a role in other pathologic processes. Although this pathway has been reported in many studies,^18^, ^19^, ^21^ evidence for its relationship with bacterial infections is still sparse. Drp1 and Mfn1 are key proteins that regulate fission ‐fusion of mitochondria. In this study, increased Drp1 expression and decreased Mfn1 expression were detected, thus suggesting a change in mitochondrial dynamics. PINK1 and Parkin were increased not only in total protein but also in mitochondrial protein, indicating that this pathway was activated. Interestingly, siRNA‐PINK1 reversed these results. However, the LC3 level did not decrease, which may be related to the compensatory enhancement of other autophagy pathways in cells. In summary, the results indicate that the mitophagy induced by *S. aureus* in bovine macrophages is PINK1/Parkin‐dependent.

Mitochondrial respiration produces ROS, and excessive ROS levels can cause damage to biological macromolecules such as proteins, nucleic acids and lipids. Considerable evidence has shown that ROS are closely related to the antibacterial or bactericidal ability of a variety of cells.^32^, ^33^ Colocalization of bacteria and mitochondria has been observed in infection,^15^, ^17^ which suggests that mtROS are more likely to exert an antibacterial effect. *S. aureus* unique cellular survival mechanism can promote intracellular survival by affecting mtROS levels.^15^, ^28^ Panton‐Valentine leukocidin (PVL) treatment has been shown to significantly increase mtROS levels.^16^ In addition, *S. aureus* infection can also trigger the production of mitochondria‐derived vesicles in a Parkin‐dependent manner, and mtROS play an antimicrobial role in the accumulation of bacteria‐containing phagosomes.^17^ The expression of catalase targeting mitochondria inhibits the clearance of *Salmonella typhimurium* by macrophages, suggesting that mtROS have bactericidal effects.^34^ Moreover, the bacterial load of *Listeria monocytogenes* in all organs of infected mice increased significantly after mtROS were specifically cleared by MitoTEMPO.^28^ In turn, bacteria have evolved efficient coping mechanisms to prevent clearance. Zhang et al. found that *L. monocytogenes* can reduce mtROS and avoid death by inducing mitophagy by releasing the toxic factor LLO.^28^ *S. aureus* can stimulate glycolysis and mtROS production in host cells in the form of small colony variants (SCVs) and induce cell necrosis unrelated to Caspase.^35^ Meanwhile, SCV cannot activate the effective antibacterial immunity of the host and “hides” in the cell.^36^ Geng et al. reported that *S. aureus* induces autophagy in bovine mammary epithelial cells and uses autophagy to survive in cells.^37^ Wang et al. also found that inhibiting the formation of autophagosomes in BMECs facilitated the clearance of intracellular *S. aureus* ^38^ Interestingly, we also found that regulating mitophagy can alter mtROS levels to influence the *S. aureus* load and cell damage in bovine macrophages. In particular, the results of interfering with PINK1 expression were consistent with those of Mdivi‐1 treatment, namely, the mtROS levels increased and bacterial load decreased. However, CCCP promotes mitophagy, and the results are contrary to those presented above. These results imply that mitophagy is closely related to the survival of intracellular *S. aureus* and the PINK1/Parkin mitophagy pathway may become a new target for the treatment of intracellular *S. aureus* infection.

## 5 CONCLUSION

We found for the first time that *S. aureus* infection of bovine macrophages causes mitochondrial damage and induces the activation of the PINK1/Parkin‐dependent mitophagy pathway. The enhancement of *S. aureus* survival in macrophages due to the PINK1/Parkin‐mediated mitophagy.

## AUTHOR CONTRIBUTIONS

**Xi Zhou:** Conceptualization (equal); data curation (lead); formal analysis (lead); investigation (lead); methodology (lead); software (lead); writing – original draft (lead). **Jianji Li:** Conceptualization (supporting); methodology (supporting). **Heng Wang:** Conceptualization (equal); funding acquisition (lead); project administration (lead); resources (lead); supervision (lead); validation (lead); writing – review and editing (lead). **Guoqiang Zhu:** Software (supporting). **Xia Meng:** Software (supporting). **jun Li:** Methodology (supporting). **Junsheng Dong:** Conceptualization (supporting); methodology (supporting). **Luying Cui:** Conceptualization (supporting); methodology (supporting). **Kangjun Liu:** Formal analysis (equal); investigation (equal); methodology (equal); software (equal).

## FUNDING INFORMATION

This research was funded by Earmarked fund for Jiangsu Agricultural Industry Technology System (No. JATS [2022]499), the Natural Science Foundation of Jiangsu Province (No. BK20211324), 333 High‐level Talent Training Project of Jiangsu Province (CN), Priority Academic Program Development of Jiangsu Higher Education Institution (PAPD), the Project D18007, and Top‐notch Academic Programs Project of Jiangsu Higher Education Institutions (TAPP).

## CONFLICT OF INTEREST

The authors declare that there is no conflict of interest regarding the publication of this article.

## Data Availability

The data that support the findings of this study are available from the corresponding author upon reasonable request.
